# The Role of diet x gene interaction in LOAD2.Plcg2^M28L^ mice

**DOI:** 10.1002/alz.091334

**Published:** 2025-01-03

**Authors:** Claudia Rangel‐Barajas, Adrian L Oblak, Cynthia M Ingraham, Christopher D Lloyd, Paul R Territo, Stacey J Sukoff Rizzo, Gregory W Carter, Michael Sasner, Gareth R Howell, Bruce T. Lamb

**Affiliations:** ^1^ 320 W 15th Street NB 110, Indianapolis, IN USA; ^2^ Stark Neuroscience Research Institute, Indianapolis, IN USA; ^3^ Indiana University School of Medicine, Indianapolis, IN USA; ^4^ Indiana University, Indianapolis, IN USA; ^5^ University of Pittsburgh School of Medicine, Pittsburgh, PA USA; ^6^ The Jackson Laboratory, Bar Harbor, ME USA; ^7^ Stark Neurosciences Research Institute, Indiana University School of Medicine, Indianapolis, IN USA

## Abstract

**Background:**

Alzheimer’s disease (AD) and other neurodegenerative diseases are typified by a robust microglial‐mediated immune response. Genetic studies have demonstrated that variants in microglial genes are linked to risk for AD. Genome‐wide association studies (GWAS) originally identified Phospholipase C gamma 2 (PLCγ2) as a novel risk gene of Alzheimer’s disease (AD). Since then, accumulating evidence from in vitro, in vivo, and human‐based studies have corroborated and extended this association, promoting Plcg2 as one of the most important risk genes of both early‐onset and late‐onset AD, harboring both common and rare risk variants with relatively large effect on AD risk.

**Method:**

To investigate the impact of Plcg2 gene expression on microglia biology and disease pathology, we have generated Plcg2^M28L^ mice on an APOE4.Trem2R47H.hAb (LOAD2) background. Cohorts of mice were established at IU and aged to 18 months on a high fat diet (HFD). Animals began the HFD at 2 months of age and were longitudinally followed to 18 months of age. At 4, 12 and 18 months, mice received an MRI and blood draws for biomarker analysis. A second cohort of animals was analyzed cross sectionally at 4, 12, 18 and 24 months of age. PET studies were completed in a third cohort. Post‐mortem studies at both sites included blood chemistry, immunohistochemistry, and molecular studies. Studies of transcriptomics and proteomics were also completed on brain tissue.

**Result:**

Utilizing our multi‐analyte approach, we are able to correlate changes in the mice and human LOAD. Overall, at 18 months there is an increase in microgliosis in male mice that is age dependent. On a high fat diet, microgliosis is observed in both male and female within the cortex, but in the subiculum, IBA1 increased only in females.

**Conclusion:**

Our findings provide evidence that Plcg2 plays an important role in LOAD and the addition of Plcg2^M28L^ to a sensitized LOAD model (LOAD2) more closely align phenotypes in the mouse to outcomes observed in human AD.

